# Comparative Evaluation of the Physiochemical Properties, and Antioxidant and Hypoglycemic Activities of *Dendrobium officinale* Leaves Processed Using Different Drying Techniques

**DOI:** 10.3390/antiox12111911

**Published:** 2023-10-26

**Authors:** Gonglin Cai, Hangmeng Dong, Shoulong Liu, Weijie Wu, Hailong Yang

**Affiliations:** 1 College of Life & Environmental Science, Wenzhou University, Wenzhou 325035, China; 21451335001@stu.wzu.edu.cn (G.C.); 21451335010@stu.wzu.edu.cn (H.D.); 21451335030@stu.wzu.edu.cn (S.L.); 2 Food Science Institute, Zhejiang Academy of Agricultural Sciences, Hangzhou 310021, China; 3 Zhejiang Provincial Key Laboratory for Water Environment and Marine Biological Resources Protection, Wenzhou University, Wenzhou 325035, China

**Keywords:** *Dendrobium officinale* leaves, drying methods, antioxidant activity, hypoglycemic activity, phenolic compounds

## Abstract

*Dendrobium officinale* leaves have the potential to be processed into natural antioxidants, functional foods, and food additives. To maximally maintain their quality, fresh *D. officinale* leaves were dehydrated using different drying methods, i.e., hot air drying (HD), microwave drying (MD), infrared drying (IRD), and freeze drying (FD), and then the physicochemical properties, microstructure, and biological activities of the dried samples were compared. The results showed that, with the FD method, the samples had a porous microstructure, maintained the highest phenolic content, and demonstrated the highest antioxidant and hypoglycemic activities. Among the three thermal drying methods, with the IRD method, the samples retained higher phenolic contents, showed stronger DPPH free-radical scavenging, ferric ion reducing, ferrous ion chelating, and α-glucosidase inhibitory abilities, and more strongly promoted glucose metabolism in insulin-resistant HL-7702 cells than the samples with the MD and HD methods. These results suggested that FD was the most suitable method. However, IRD might be a promising alternative, owing to the high cost and long time needed for FD for the large-scale drying of *D. officinale* leaves.

## 1. Introduction

*Dendrobium officinale*, known as ‘TiepiShihu’ in Chinese, is a famous edible and medicinal plant used to treat fever, moisten the lungs, and nourish the stomach for thousands of years [1,2]. A wild resource of *D. officinale* is rare because its growth needs a special habitat and is very slow. In China, with the breakthrough of artificial planting techniques, *D. officinale* has been commercially cultivated in Zhejiang, Guizhou, Guangxi, Yunnan, and other provinces of China, and the planting area exceeds 13,000 ha nowadays [3].

Traditionally, only *D. officinale* stems are collected as the main products, while the leaves are treated as by-products [2,4]. The leaves account for 50% of the total biomass of *D. officinale*. Therefore, the amount of *D. officinale* leaves is considerable [2]. Presently, only a small amount of leaves are processed into *D. officinale* leaf tea after impurity removal, cleaning, drying, and other steps [3], while a large amount of *D. officinale* leaves are discarded [4]. Modern research indicates that the leaves of *D. officinale* contain bioactive constituents similar to those in the stem, including polysaccharides, flavonoids, alkaloids, and bibenzyl compounds, and have multi-biological activities such as antioxidant, hypoglycemic, and immunoregulatory activities, gut microbiota regulation, etc. [2,4,5,6]. Therefore, *D. officinale* leaves have the potential to be processed into natural antioxidants, functional foods, and food additives [3].

The fresh leaves of *D. officinale* have a high moisture content (about 90%) [3], which makes the plant matrix perishable and susceptible to microbial growth, thereby limiting their storage, transport, and further processing [7,8]. Drying is an efficient method to prolong the storage time and prevent the loss of bioactive ingredients in post-harvest *D. officinale* leaves [3], and hot air drying (HD) is the most commonly used method in large-scale drying. In addition, freeze drying (FD), microwave drying (MD), and other techniques have also been developed for *D. officinale* leaf dehydration. However, different methods may lead to differences in drying efficiency, quality, and biological activity of the dried leaves. For example, Chen et al. [7] found MD to be the most suitable method to preserve bioactive (crocins) and aroma components in *saffron* after comparing different drying techniques. However, Saikia et al. [8] reported that FD is the most efficient method to preserve the post-harvest qualities and essential oils of *Alpinia zerumbet* rhizome. For the drying of *Stevia rebaudiana* leaves, Lemus-Mondaca et al. [9] concluded that FD is better for maintaining polyphenols and antioxidant capacity, infrared drying (IRD) can preserve high antimicrobial activity, and MD is better for retaining anti-inflammatory activity. As for *D. officinale* leaves, they have a thick cuticle on their leaf surface, which may hinder the diffusion of water from the inside to the outside during the drying process. Therefore, it is crucial to choose suitable dehydration methods to enhance the drying efficiency and preserve the bioactive ingredients and bioactivities of *D. officinale* leaves.

In this work, *D. officinale* leaves were treated using four different drying processes: MD, HD, IRD, and FD. Subsequently, their physicochemical properties, antioxidant capacity, protective activity against DNA oxidation, and hypoglycemic activity were compared. The results provide necessary information on obtaining dried *D. officinale* leaves of high quality.

## 2. Materials and Methods

### 2.1. D. officinale Leaves and Drying

Fresh *D. officinale* leaves were collected from Yueqing Lvfeng TiepiShihu Planting Cooperative (Wenzhou, China) in May 2022. Leaves with mechanical damage and blemishes were picked out, and those with similar appearances (color and size) were collected for experiments.

The samples were dried using the MD, HD, IRD, and FD methods. Based on the preliminary experiment, the MD method was performed in a microwave oven (G70F23CN2P; Galanz Group, Foshan, China) at 420 W. The IRD method was conducted at 55 °C in a mini-type infrared dryer (M3125; Minghong Machinery Factory, Zhoushan, China). The HD method was performed at 55 °C in an experimental drying oven (DGG-9240A; Shengxin Scientific Instrument Co., Ltd., Shanghai, China). The samples were subjected to FD in a freeze dryer (LyoQuest-85; Telstar Technologies Co., Ltd., Barcelona, Spain) after being frozen at −70 °C for 24 h, and the condenser temperature, vacuum pressure, and plate temperature were set at −80 °C, 0.05 mBar, and 25 °C, respectively. During the drying process, sampling was performed at regular intervals. Additionally, the samples were weighed, and their moisture content was calculated. All drying methods were performed until the moisture content of the *D. officinale* leaves was below 10%, and each drying method was performed in triplicate. For the chemical and biological activity determination, dried *D. officinale* leaves were pulverized, sieved, and stored in a refrigerator at −20 °C.

### 2.2. Moisture Content, Rehydration Ratio, and Color Measurements

The moisture content of *D. officinale* leaves was obtained gravimetrically.

Rehydration was performed by dipping 1.0 g of dried sample into 25 mL of deionized water at 40 °C for 4 h. The rehydration ratio was calculated from the weight of the *D. officinale* leaves before and after rehydration.

The color of the *D. officinale* leaves was recorded by a Konica chroma meter (CR-5, Minolta, Tokyo, Japan) using L* (lightness/darkness), a* (redness/greenness), and b* (yellowness/blueness) values. The color changes (∆E) between the fresh and dried *D. officinale* leaves were obtained using the following equation [10].

$$∆E=\sqrt{{\left(L^{*}-L_{0}^{*}\right)}^{2}+{\left(a^{*}-a_{0}^{*}\right)}^{2}+{\left(b^{*}-b_{0}^{*}\right)}^{2}}$$

where $a_{0}^{*}$, $b_{0}^{*}$, and $L_{0}^{*}$ are the values of the fresh samples, and *a*^∗^, *b*^∗^, and *L*^∗^ are the values of the dried samples.

### 2.3. Microstructure Observation

Dried *D. officinale* leaves were transversely cut, and the leaf transection was sputtered with gold using a JFC-1600 sputter coater (JEOL, Tokyo, Japan) under a vacuum. Subsequently, the microstructure of the leaves’ cross-section was analyzed at a magnification of 250 and 1000 using a NanoSEM200 scanning electron microscope (FEI, Hillsboro, OR, USA).

### 2.4. Phenolic Compound Content Measurements

Dried *D. officinale* leaves were extracted as described by Si et al. [11] with slight modifications. Briefly, 1.0 g of sample was extracted with 40 mL of 70% ethanol at 60 °C for 1 h, centrifuged, and the supernatant was collected. The individual phenolic compound contents were analyzed by HPLC (1260 Infinity II; Agilent Technologies, Santa Clara, CA, USA), equipped with a ZORBAX Eclipse Plus C18 column (5 μm, 4.6 × 250 mm), using a gradient elution of 0.1% formic acid (solution A) and acetonitrile (solvent B) at 35 °C and a flow rate of 0.5 mL min^−1^. The injection volume was 10 μL, and the elution was performed as follows: 5% B (0~4 min), 5% to 40% B (5~34 min), 40% B (34~40 min), 40% to 5% B (40~42 min), and 5% B (42~50 min). The detection absorbance was set at 254 nm and 330 nm, respectively [10].

### 2.5. Measurements of Total Polysaccharide and Uronic Acid Contents

Two grams of samples were added into 60 mL of deionized water and extracted twice at 70 °C for 2 h. The supernatant was combined and concentrated under a vacuum. The crude polysaccharide was obtained by adding ethanol (4 times), leaving it overnight, and washing it with ethanol (80%). Subsequently, the precipitation was dissolved in deionized water and analyzed using the phenol–sulfuric acid method [5].

For the uronic acid content determination, the crude polysaccharides were dialyzed using a 3500D dialysis bag in running water for 48 h and then lyophilized. The uronic acid content in the crude polysaccharides was measured using the m-hydroxybiphenyl method with D-galacturonic acid as a standard [12].

### 2.6. Antioxidant Activity Measurements

The samples were extracted as described in 2.4, and their antioxidant activity was evaluated by the following four different assays: scavenging activities against 1,1-diphenyl-2-picrylhydrazyl (DPPH) and 2,2′-azinobis (3-ethylbenzothiazoline-6-sulfonic acid) (ABTS) free radicals, reducing power on ferric ions, and metal chelating activity on ferrous ions.

The scavenging activities against DPPH and ABTS free radicals of the samples were performed as described in our previous research [10,13], and the results were expressed as mg of ascorbic acid equivalents per gram of dried sample (mg AsAE g^−1^). The ability of the samples to reduce Fe^3+^ was determined using the potassium ferricyanide method, as described by Li et al. [14], and the results were expressed as mg of ascorbic acid equivalents per gram of dried sample (mg AsAE g^−1^). The chelating Fe^2+^ ability of the samples was determined using the ferrozine method, as described by Zengin et al. [15], and the results were expressed as mg of EDTA equivalents per gram of dried sample (mg EDTAE g^−1^).

### 2.7. DNA Damage Protective Activity Measurement

Dried *D. officinale* leaves were extracted as described in 2.4. For the DNA damage protective activity measurement, 1 μL of pBR322 plasmid DNA (0.5 μg μL^−1^), 5 μL of extract, 10 μL of PBS buffer (pH 7.2), and 4 μL of Fenton’s reagent (1 mmol L^−1^ FeSO_4_ and 1 mmol L^−1^ H_2_O_2_) were mixed and reacted at 25 °C for 30 min. Subsequently, electrophoresis was performed on 0.8% agarose at 120 V. DNA was observed and photographed under ultraviolet light using a gel-imaging system after being stained with ethidium bromide. Rutin (100 μg mL^−1^) and PBS were used as the positive and negative controls, respectively. The intensity of the DNA band in the photograph was analyzed using the ImageJ software-win64 (National Institutes of Health, Bethesda, MD, USA) and used to calculate the percentage of supercoiled DNA (undamaged DNA) in each gel lane.

### 2.8. Enzyme Inhibitory Ability Measurements

Dried *D. officinale* leaves were extracted as described in 2.4. Subsequently, the collected supernatant was evaporated under vacuum to dry and dissolved in DMSO (dimethyl sulfoxide) for an enzyme inhibitory ability analysis [10].

The inhibition capacity of dried *D. officinale* leaves against porcine pancreatic α-amylase was assayed with reference to the method of Zengin et al. [16]. Briefly, starch was hydrolyzed by porcine pancreatic α-amylase (22.2 U mL^−1^; Sigma-Aldrich, St. Louis, MO, USA) at 37 °C for 10 min and terminated by adding HCl (2 mol L^−1^). The residual starch content was determined using the I-KI reagent at 660 nm. The inhibition ability was obtained by comparing the amount of hydrolyzed starch between the test sample and control (0.025 mmol L^−1^ PBS; pH 7.0). The result was calculated and presented based on the dried sample using acarbose (Aladdin, Shanghai, China) as a reference drug (mg ACE g^−1^).

The inhibition ability of dried *D. officinale* leaves against α-glucosidase was assayed in 96-well plates with reference to the method of Bai et al. [17]. Briefly, 4-nitrophenyl-α-D-glucopyranoside (4 mmol L^−1^, Thermo, Waltham, MA, USA) was hydrolyzed by α-glucosidase (0.2 U mL^−1^; Sigma-Aldrich, USA) at 37 °C for 30 min and then terminated by adding Na_2_CO_3_ (1 mol L^−1^). The 4-nitrophenol produced was determined at 405 nm using a microplate reader. The inhibition ability was calculated by comparing the amount of produced 4-nitrophenol between the test sample and control (0.025 mmol L^−1^ PBS; pH 6.8) and presented based on the dried sample using acarbose as a standard (mg ACE g^−1^).

### 2.9. Cell Experiments

Samples were prepared as described in 2.7, dissolved in DMSO, and diluted to different concentrations using RPMI-1640. Human hepatocyte HL-7702 cells were cultured in RPMI-1640 containing 10% FBS and 1% penicillin/streptomycin under the conditions of 37 °C and 5% CO_2_. The cells were pipetted into the wells of 96-well plates (5000 cells per well) and cultured until the cells adhered to the wall. Subsequently, the media were replaced with media containing the test samples (12.5~400 μg mL^−1^) and cultured for 24 h. Media containing DMSO were used as a control. The cell viability was determined using the MTT assay kit (Beyotime Biotechnology, Shanghai, China).

For the hypoglycemic activity analysis, cell culture was performed in 6-well plates (2.0 × 10^5^ cells per well). The insulin-resistant cell model was established by referencing the method of Fan et al. [18]. Briefly, the attached cells were starved in serum-free RPMI-1640 for 12 h and then transferred into high-glucose (36.11 mM) media containing 100 nM insulin and incubated for 24 h. Normal cells were incubated in media containing 11.11 mM glucose. For the hypoglycemic activity analysis, the samples (12.5~50 μg mL^−1^) were added to insulin-resistant cells and incubated for 12 h. Metformin (400 μM) was used as a positive control. After incubation, the culture media were collected for a glucose concentration assay using a commercial kit (Beyotime Biotechnology, Shanghai, China). The cells were washed with PBS three times, detached from the plate, and collected by centrifugation. The glycogen content and phosphoenolpyruvate carboxykinase (PEPCK) activity were determined using commercial kits purchased from the Nanjing Jiancheng Bioengineering Institute (Nanjing, China). The glucose-6-phosphatase (G6P) activity was determined using a commercial kit purchased from Beyotime Biotechnology (Shanghai, China).

### 2.10. Data Analysis

All experiments were performed at least three times, and the results were expressed as a mean ± standard deviation. A one-way analysis of variance (ANOVA) was performed with Duncan’s multiple range tests using the SPSS 23.0 software, and the statistical significance was set at *p* < 0.05.

## 3. Results and Discussion

### 3.1. Drying Time, Color, Microstructure, and Rehydration

The moisture content of fresh *D. officinale* leaves was measured to be 89.70 ± 0.54%. To limit their biochemical metabolism and exogenous microbial growth, the moisture content of the samples must be less than 10% [19]. After the drying procedures, the moisture of *D. officinale* leaves decreased gradually, and the time was 0.68 h, 37.50 h, 66.82 h, and 127.16 h for MD, IRD, HD, and FD, respectively, to reach the moisture standard of less than 10%. The drying time was much longer than for other agricultural products, such as edamame [20], purple basil leaves [21], *C. comatus* [13], etc. That is owing to the thicker cuticle of *D. officinale* leaves, which can prevent water diffusion [22], resulting in much longer drying times.

During the drying process, the Maillard reaction, enzymatic darkening, oxidation, and pigment degradation may cause color changes in agricultural products [23]. As presented in Figure S1, the color of the *D. officinale* leaves was markedly changed by some of the thermal drying methods (HD, IRD, and MD). Correspondingly, the color parameters (L*, a*, and b*) were significantly (*p* < 0.05) changed by the HD, IRD, and MD methods, while the FD method only significantly changed the value of a* (Table 1). It was more suitable to evaluate the color change using a combination of L*, a*, and b*. As presented by the values of ∆E, the MD method caused the maximum color change between the dried and fresh samples, followed by the IRD and HD methods, while the FD method caused the least change due to the fact that the conditions of the FD method were hypoxia and a lower temperature [24].

**Figure 1:**
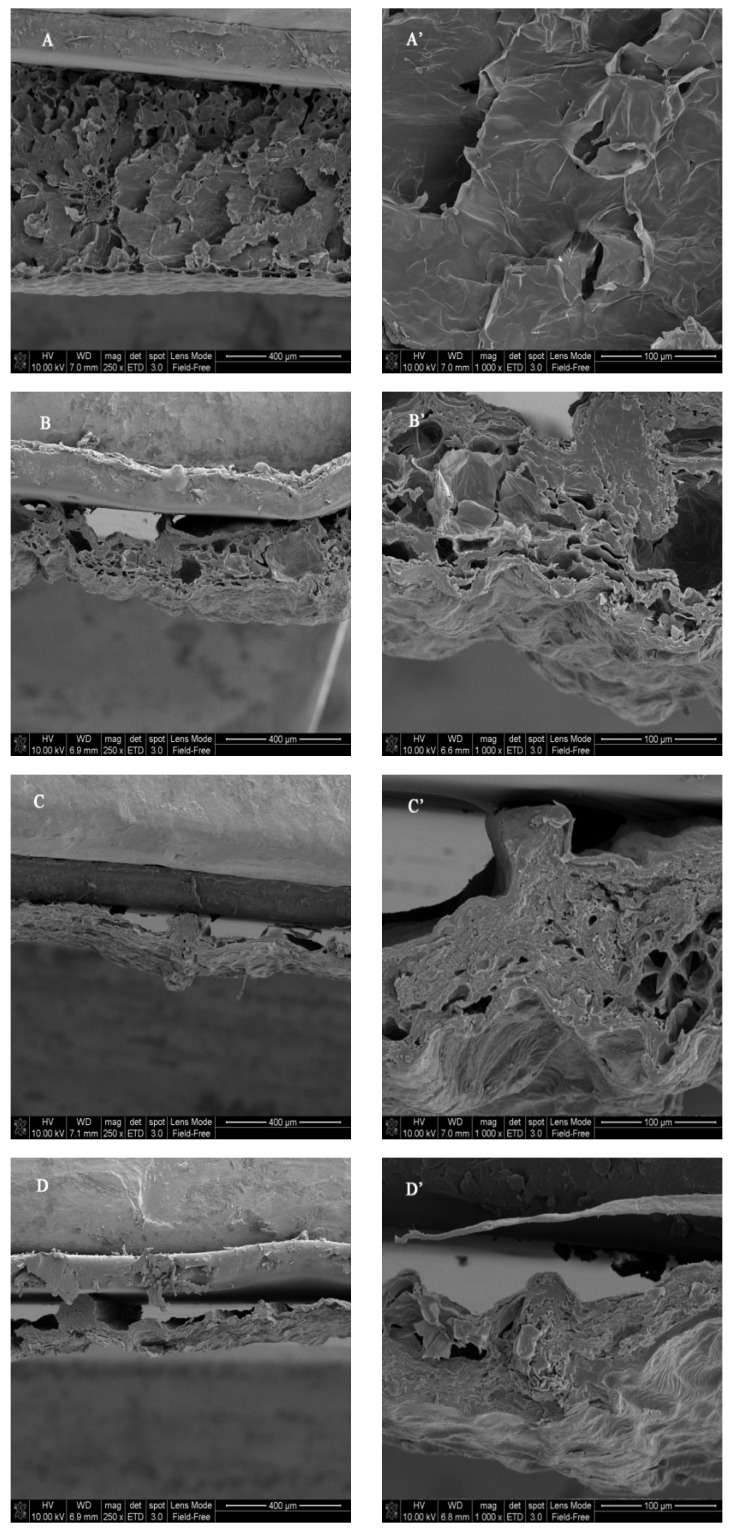
Microstructure of *Dendrobium officinale* leaves dried by different methods. (**A**–**D**) are the scanning electron micrographs of samples dried by freeze drying (FD), infrared drying (IRD), hot air drying (HD), and microwave drying (MD), respectively, at a magnification of 250. (**A’**–**D’**) are the samples at a magnification of 1000.

Dehydration may also lead to variations in the overall shape and tissue microstructure of *D. officinale* leaves. As shown in Figure 1, the FD method (Figure 1A,A’) created a porous microstructure with thin hole walls because it removed water directly from the frozen phase under vacuum [19], thereby maintaining the original structure of the cells and tissues, which is preferred. The IRD (Figure 1B,B’) and HD (Figure 1C,C’) methods also created porous microstructures in which the holes were less and distributed more irregularly than those in the FD sample. Comparatively, there were more holes in the IRD sample than in the HD sample. The MD method (Figure 1D,D’) created dense microstructures with some large cavities due to the irregular collapse and shrinkage of the cellular tissues.

Rehydration capacity is one of the indices related to the physical and chemical variations of dried agricultural products, and it can be used to evaluate their microstructure alterations [20]. As presented in Table 1, the FD sample had the highest rehydration ratio of 5.59, followed by the IRD (4.03) and HD (3.74) samples, while the MD sample showed the lowest ratio (3.63). The results were consistent with the microstructures of the dried *D. officinale* leaves presented in Figure 1. The rehydration capacity of the dried *D. officinale* leaves was proportional to their porous microstructures, whereas dense microstructures caused difficulty in water absorption [10].

### 3.2. Phenolic Compounds

**Figure 2:**
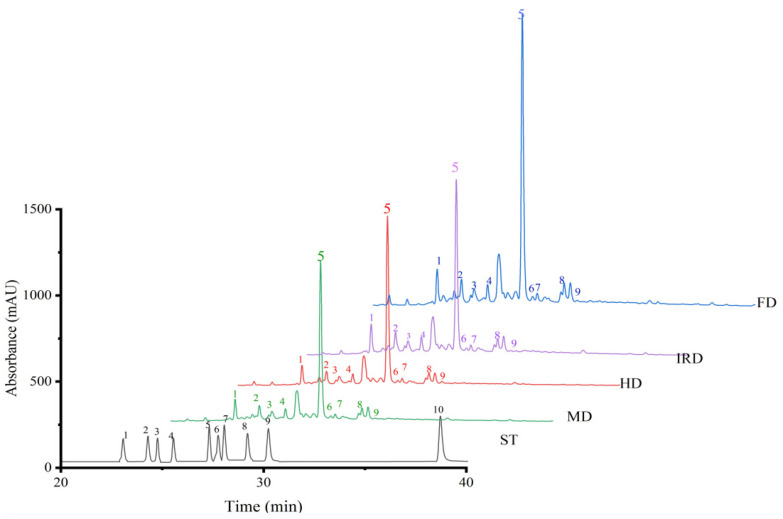
Phenolic profiles of *Dendrobium officinale* leaves dried by different methods. FD—freeze drying; IRD—infrared drying; HD—hot air drying; MD—microwave drying; ST—standards. 1, vicenin II; 2, vicenin I; 3, schaftoside; 4, isoschaftoside; 5, rutin; 6, isovitexin; 7, apigenin 6-C-α-L-arabinopyranosyl-8-C-β-D-xylopyranoside; 8, kaempferol-3-rutinoside; 9, ferulic acid; 10, quercetin.

The main phenolic compounds in *D. officinale* leaves include rutin, vicenin I, vicenin II, schaftoside, isoschaftoside, isovitexin, kaempferol-3-rutinoside, etc. [2,3,25]. To explore the effect of the drying techniques on the phenolic compounds of *D. officinale* leaves, the phenolic profiles were analyzed by HPLC, and the results are shown in Figure 2 and Table 2. Consistent with the results of Zhang et al. [2], rutin was determined to be the dominant phenolic compound in *D. officinale* leaves, followed by vicenin II, vicenin I, schaftoside, and isoschaftoside, while the aglycone of rutin (quercetin) was not detected due to its low content in crude extracts.

For all of the detected phenolic monomers, non-thermal drying resulted in significantly (*p* < 0.05) higher contents than with the thermal drying methods (Table 2) due to the fact that FD was operated under the conditions of low temperature and hypoxia, which can result in lower thermal degradation and oxidation for phenolic compounds, thereby showing higher phenolic reservation [26]. Similar results were also determined for the FD of *Angelica keiskei* [19] and *S. fusiforme* [10]. However, Li et al. [27] reported that the HD method was more suitable for phenolic preservation than the FD method. The negative or positive impacts of drying methods on phenolic ingredients might be determined by the drying materials, individual phenolic monomers, and their location in the cell [10]. For the thermal drying of *D. officinale* leaves, the IRD method showed a significantly (*p* < 0.05) higher content of all the detected phenolic monomers compared with the HD and MD methods (Table 2).

### 3.3. Polysaccharides and Their Uronic Acid Content

**Figure 3:**
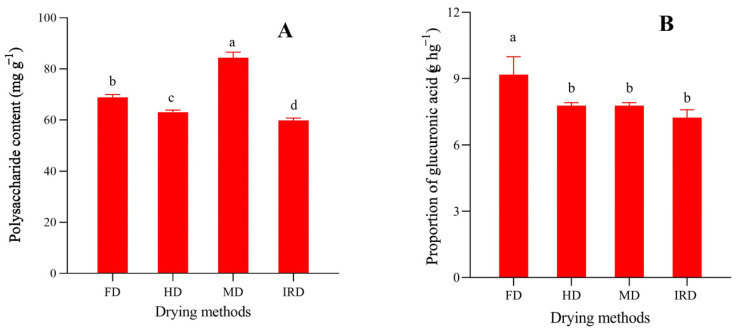
Effects of drying methods on polysaccharide (**A**) and uronic acid (**B**) contents in dried *Dendrobium officinale* leaves. Significant differences at *p* < 0.05 are indicated by different letters. FD—freeze drying; IRD—infrared drying; HD—hot air drying; MD—microwave drying.

A polysaccharide is another bioactive compound that exists in *D. officinale* leaves that has been shown to be involved in immunoregulation [28], gut microbiota regulation [6], anti-inflammatory [29], and hypoglycemic [5] activities. As shown in Figure 3A, the content of polysaccharides in dried *D. officinale* leaves ranged from 59.78 to 84.38 mg g^−1^, among which the MD sample had the highest one. Previous studies also showed similar results in dried *S. fusiforme* [10], *Ganoderma lucidum* [30], and *C. comatus* [13]. These results might be due to the fact that the MD method causes high temperatures on the inside of plant materials, resulting in higher melanoidin contents and/or more polysaccharide release from cells [10].

Uronic acid, including galacturonic acid [29] or glucuronic acid [5], is one of the main monosaccharide compositions in polysaccharides extracted from *D. officinale* leaves. It was reported that the uronic acid content has a strong correlation with the biological activity of polysaccharides [31], and drying techniques may significantly affect the uronic acid content of polysaccharides [12,31]. The uronic acid content was determined to be 7.24 g hg^−1^, 7.78 g hg^−1^, 7.93 g hg^−1^, and 9.17 g hg^−1^ in polysaccharides from *D. officinale* leaves dried using the IRD, HD, MD, and FD methods, respectively (Figure 3B). Undoubtedly, the FD sample had the highest polysaccharide content. The result was in accordance with the results of Fu et al. [12] in loquat leaf drying, Yan et al. [32] in bitter gourd slice drying, and An et al. [31] in lychee pulp drying, owing to the lower glucuronidase activity in the FD process.

### 3.4. Antioxidant Activity

Antioxidant activity is one of the main biological activities of *D. officinale* leaves due to their high flavonoid and polysaccharide contents [2,4]. Different drying methods may lead to differences in the antioxidant activity of dried plant leaves, such as *Hibiscus cannabinus* leaves [26], *Cichorium intybus* leaves [27], *L. petersonii* leaves [8], etc. In this study, the effects of the FD, MD, HD, and IRD methods on the antioxidant activity of *D. officinale* leaves were evaluated by free-radical scavenging, reducing power, and metal chelating activities and the results are shown in Table 2. Among the samples dried by thermal drying techniques, the IRD sample showed higher DPPH radical scavenging ability than the MD (*p* > 0.05) and HD (*p* < 0.05) samples. The IRD sample also showed higher ferric ion-reducing power than the HD (*p* > 0.05) and MD (*p* < 0.05) samples. The HD sample showed significantly (*p* < 0.05) higher ABTS radical scavenging ability than the IRD and MD samples (with insignificant differences between them). No significant (*p* > 0.05) differences were observed for the ferrous ion chelating activity among the samples dried by the IRD, MD, and HD methods. Compared with the samples dehydrated by thermal drying techniques, the FD sample showed the highest DPPH and ABTS radical scavenging ability, ferric ion reducing power, and ferrous ion chelating activity, with significant differences (*p* < 0.05). Additionally, similar results were observed for drying technique research on *H. cannabinus* leaves [26], *L. petersonii* leaves [33], *A. keiskei* [19], *S. fusiforme* [10], etc. The antioxidant activity of *D. officinale* leaves is highly correlated with their phenolics, and the correlation coefficients between total phenolics and DPPH free-radical scavenging ability, ferric ion reducing power, and ferrous ion chelating activity were 0.863, 0.940, and 0.936, respectively.

### 3.5. Protective Activity against DNA Oxidation

**Figure 4:**
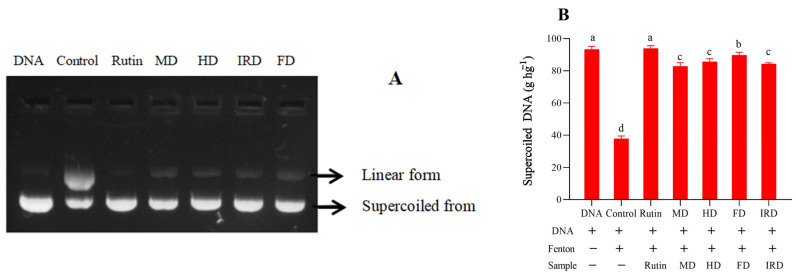
Effects of freeze drying (FD), microwave drying (MD), infrared drying (IRD), and hot air drying (HD) on the protective activity of dried *Dendrobium officinale* leaves against DNA oxidative damage. (**A**), electrophoretogram (DNA: PBS + DNA. Control: DNA + Fenton’s reagent. Rutin: rutin + DNA + Fenton’s reagent. MD: MD sample + DNA + Fenton’s reagent. HD: HD sample + DNA + Fenton’s reagent. FD: FD sample + DNA + Fenton’s reagent. IRD: IRD sample + DNA + Fenton’s reagent). (**B**), percentage of supercoiled DNA. Significant differences at *p* < 0.05 are indicated by different letters. FD—freeze drying; IRD—infrared drying; HD—hot air drying; MD—microwave drying.

*D. officinale* leaves are rich in phenolics and other bioactive compounds and have strong protective activity against oxidation [2,4]. Previous research showed that drying methods might affect the protective activities of plant matrices against DNA damage, such as in cumin essential oil [34] and *S. fusiforme* [10]. The effects of *D. officinale* leaves dried by different methods on DNA oxidative damage protection were also measured. As shown in Figure 4A, DNA was converted from a supercoiled form into open circular and linear forms after adding Fe^2+^ and H_2_O_2_. The extractions from *D. officinale* leaves showed a protective effect for supercoiled DNA to some extent, and the FD sample showed the best protective effect. The intensity percentage of the supercoiled DNA maintained by the FD sample was 89.78 g hg^−1^, which is significantly (*p* < 0.05) higher than those presented by the other three samples. Additionally, the difference in DNA damage protective activity among the HD, MD, and IRD samples was insignificant (Figure 4B).

### 3.6. Inhibition Activities against α-Amylase and α-Glucosidase

In recent decades, the occurrence of diabetes mellitus has increased, and one of the treatment strategies is to inhibit the key enzymes linked to the disease [15]. Assays of inhibition against key enzymes for carbohydrate digestion (α-glycosidase and α-amylase) have been widely used to estimate the hypoglycemic potential of plant extracts in vitro [31]. Traditionally, *Dendrobium* leaves have been used to treat metabolic syndromes [4]. In this study, the effects of *D. officinale* leaves dried by different methods on the activities of α-glucosidase and α-amylase were determined and are shown in Table 2. Statistical analysis showed that the inhibitory activity against α-amylase was ranked as follows: FD > IRD/MD > HD. The FD method showed significantly (*p* < 0.05) higher activity, followed by the IRD or MD methods (with insignificant differences between them), while the HD sample presented significantly (*p* < 0.05) lower activity. The inhibitory activity against α-glucosidase was significantly (*p* < 0.05) different for the samples dried by the different methods, with a rank of FD > IRD > MD > HD. Drying methods affecting the enzyme inhibition activities against α-glucosidase and α-amylase were also reported in other plant matrices, such as lychee pulp [31] and Chinese bayberry pulp [35], owing to differences in the contents of phenolic and/or polysaccharide ingredients.

### 3.7. Glucose Metabolism in HL-7702 Cells

**Figure 5:**
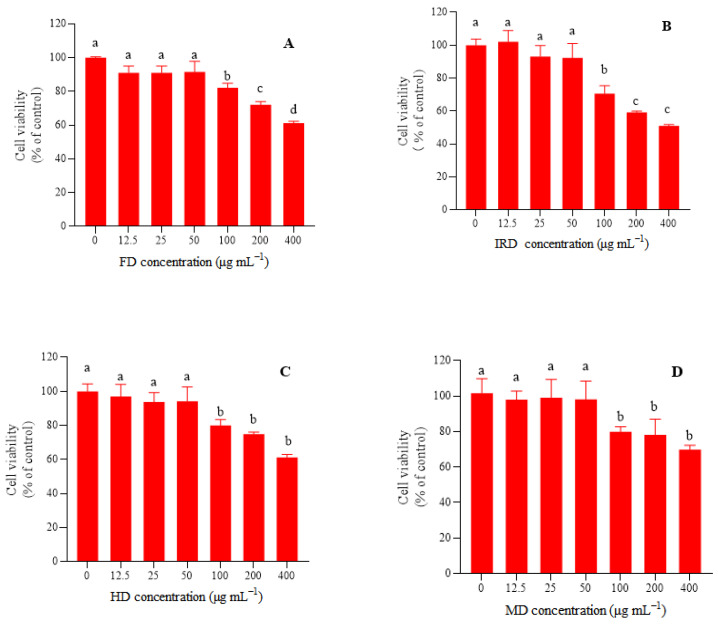
Effect of extracts of *Dendrobium officinale* leaves on cell viability of HL-7702 cells. Significant differences at *p* < 0.05 are indicated by different letters. (**A**), freeze drying (FD) sample; (**B**), infrared drying (IRD) sample; (**C**), hot air drying (HD) sample; (**D**), microwave drying (MD) sample.

Drying may change the contents and constituents of bioactive compounds, resulting in an alternation in the hypoglycemic activity of plant medicines, which has been verified not only by enzyme inhibition experiments [35] but also by cell and mouse experiments [16]. In vitro and in vivo experiments showed that hypoglycemic activity is an important bioactivity of *D. officinale* leaves due to their high phenolic and polysaccharide contents, and one of the hypoglycemic mechanisms of *D. officinale* leaves is to regulate glucose metabolism [2,36]. In this study, the effects of the FD, MD, HD, and IRD methods on the hypoglycemic activity of *D. officinale* leaves were also evaluated by glucose consumption, glycogen synthesis, and gluconeogenesis-related key enzymes in HL-7702 cells. As shown in Figure 5, the extracts from *D. officinale* leaves dried by different methods showed similar effects on cell viability. There was no significant (*p* > 0.05) inhibitory effect on the HL-7702 cells at a low dose (<50 μg mL^−1^), while a significant (*p* < 0.05) toxic effect was observed at 100 μg mL^−1^. Therefore, 50 μg mL^−1^ was set as the dose for all samples for the glucose metabolism experiment.

**Figure 6:**
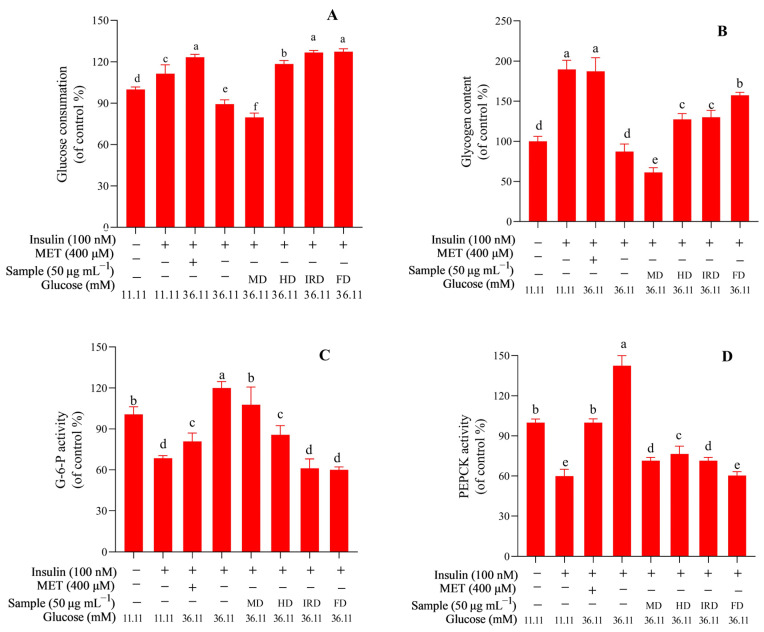
Effect of extracts of *Dendrobium officinale* leaves on the glucose consumption (**A**), glycogen content (**B**), G-6-P activity (**C**), and PEPCK activity (**D**) of HL-7702 cells. Significant differences at *p* < 0.05 are indicated by different letters. FD—freeze drying; IRD—infrared drying; HD—hot air drying; MD—microwave drying.

As shown in Figure 6A, the glucose consumption of insulin-resistant cells was significantly (*p* < 0.05) lower than that of normal cells, but it could be effectively reversed after treatment with extracts from *D. officinale* leaves dried by the FD, IRD, and HD methods in the order of FD > IRD > HD (with insignificant differences between the FD and IRD methods). However, the MD methods did not have this effect. Improvement in hepatic glucose consumption by some natural extracts can lead to enhanced glycogen synthesis [37]. Compared with that of normal cells, a significant (*p* < 0.05) increase in glycogen content of insulin-resistant cells was achieved via treatment with extracts from *D. officinale* leaves dried by the FD, IRD, and HD methods, and the increase order was FD > IRD > HD. However, the MD group did not have the same increase (Figure 6B). Gluconeogenesis is an important process in glucose metabolism, and PEPCK and G6P are key enzymes in hepatic gluconeogenesis [18]. Gluconeogenesis is increased in insulin-resistant hepatocytes after high-glucose exposure, and some natural compounds/extracts can lower gluconeogenesis by inhibiting the activities of PEPCK and G6P, such as mangiferin derivatives [18], citrus flavonoids [38], *Dendrobium nobile* extract [39], etc. As shown in Figure 6C,D, the extracts from *D. officinale* leaves could also significantly (*p* < 0.05) decrease the PEPCK and G6P activities of insulin-resistant HL-7702 cells, while their inhibitory effect was affected by the drying methods. The inhibitory activity against G6P was in the order of FD > IRD > HD > MD (with insignificant differences between the FD and IRD methods), while against PEPCK, it was in the order of FD > IRD > MD > HD (with insignificant differences between the IRD and MD methods).

## 4. Conclusions

In conclusion, inevitable structural and color changes occurred in *D. officinale* leaves during the use of the drying techniques, and the FD sample showed less color change, a more porous microstructure, and a higher rehydration ratio. Among the four drying techniques, the FD sample retained the highest phenolic content, resulting in the highest antioxidant, DNA damage protective, and hypoglycemic activities. The IRD sample showed the second-highest rehydration ratio, phenolic content, DPPH radical scavenging, Fe^3+^ reducing, and hypoglycemic activities. The MD sample maintained the highest polysaccharide content and second-highest α-amylase inhibitory activity. The HD sample had the second-highest DNA damage protective abilities. Overall, FD was the most suitable method for obtaining dried *D. officinale* leaves with good appearance, high phenolic content, and high biological activity. However, it exhibited the lowest drying efficiency. The thermal drying methods (MD, HD, and IRD) showed a much higher drying efficiency, and the IRD sample was comprehensively better in terms of phenolics and biological activity than the HD and MD samples. The FD method might be suitable for the drying of *D. officinale* leaves on a small scale for producing products of high quality. The IRD method could be an alternative technique for the large-scale drying of *D. officinale* leaves.

## Figures and Tables

**Table 1:** Color parameters and rehydration ratio of *Dendrobium officinale* leaves dried by different methods.

Drying Method	L*	a*	b*	ΔE	Rehydration Ratio
Fresh	30.49 ± 0.14 a	−4.28 ± 0.25 e	13.69 ± 0.88 a	-	-
HD	25.38 ± 0.14 b	2.17 ± 0.10 c	12.07 ± 0.19 b	8.43 ± 0.32 c	3.74 ± 0.10 c
MD	19.60 ± 0.77 d	2.55 ± 0.15 b	5.43 ± 0.11 d	15.31 ± 0.55 a	3.63 ± 0.21 d
IRD	22.89 ± 0.22 c	3.43 ± 0.08 a	11.48 ± 0.28 c	11.09 ± 0.05 b	4.03 ± 0.14 b
FD	30.52 ± 0.85 a	−1.46 ± 0.17 d	13.27 ± 0.35 a	2.95 ± 0.07 d	5.59 ± 0.07 a

Significant differences at *p* < 0.05 in the index (each column) are indicated by different letters. FD—freeze drying; IRD—infrared drying; HD—hot air drying; MD—microwave drying.

**Table 2:** Main phenolic monomers and antioxidant and enzyme inhibitory activities of *Dendrobium officinale* leaves dried by different methods.

Assays	Drying Methods			
	HD	IRD	MD	FD
Rutin (μg g^−1^)	2311.96 ± 4.35 c	2516.91 ± 7.07 b	2375.84 ± 103.38 c	4267.30 ± 21.51 a
Vicenin II (μg g^−1^)	298.84 ± 0.52 c	438.66 ± 1.32 b	267.30 ± 9.37 d	540.49 ± 2.18 a
Vicenin I (μg g^−1^)	195.74 ± 4.10 c	302.26 ± 2.46 b	170.24 ± 0.49 d	375.54 ± 11.58 a
Schaftoside (μg g^−1^)	179.58 ± 0.75 c	236.45 ± 1.84 b	163.88 ± 11.50 d	295.83 ± 4.22 a
Isoschaftoside (μg g^−1^)	171.21 ± 0.27 c	255.67 ± 1.24 b	153.94 ± 3.90 d	310.63 ± 1.81 a
Isovitexin (μg g^−1^)	32.01 ± 0.20 d	39.93 ± 0.45 b	33.78 ± 1.19 c	60.64 ± 0.79 a
Apigenin 6-C-α-L-arabinopyranosyl-8-C-β-D-xylopyranoside (μg g^−1^)	52.33 ± 0.20 d	73.83 ± 0.19 b	55.04 ± 1.59 c	91.28 ± 0.51 a
Kaempferol-3-rutinoside (μg g^−1^)	42.94 ± 0.47 c	54.31 ± 0.90 b	40.68 ± 0.85 c	112.36 ± 10.15 a
Ferulic acid (μg g^−1^)	2.45 ± 0.18 d	7.06 ± 0.16 b	3.67 ± 0.37 c	11.17 ± 0.16 a
Quercetin (μg g^−1^)	Nd	Nd	Nd	Nd
Total (μg g^−1^)	3287.06 ± 11.04	3925.08 ± 15.63	3264.37 ± 132.64	6065.24 ± 52.91
DPPH radical scavenging (mg AsAE g^−1^)	5.69 ± 0.08 c	6.44 ± 0.07 b	6.43 ± 0.11 b	7.26 ± 0.12 a
ABTS radical scavenging (mg AsAE g^−1^)	7.13 ± 0.02 b	6.13 ± 0.16 c	6.06 ± 0.09 c	7.43 ± 0.19 a
Reducing Fe^3+^ power (mg AsAE g^−1^)	0.88 ± 0.01 b	0.90 ± 0.01 b	0.85 ± 0.01 c	0.98 ± 0.02 a
Chelating Fe^2+^ ability (mg EDTAE g^−1^)	66.26 ± 2.02 b	65.37 ± 1.15 b	67.29 ± 0.66 b	83.65 ± 2.05 a
α-amylase inhibitory (mg ACE g^−1^)	13.32 ± 0.33 c	14.14 ± 0.06 b	14.36 ± 0.14 b	17.65 ± 0.48 a
α-glucosidase inhibitory (mg ACE g^−1^)	3.83 ± 0.46 d	25.33 ± 2.55 b	8.44 ± 1.01 c	44.87 ± 1.93 a

Significant differences at *p* < 0.05 in each index (each row) are indicated by different letters. Nd—not detected. FD—freeze drying; IRD—infrared drying; HD—hot air drying; MD—microwave drying.

## Data Availability

The data are contained within the article.

## Supplementary Materials

The following supporting information can be downloaded at: https://www.mdpi.com/article/10.3390/antiox12111911/s1, Figure S1. Photos of fresh and dried *Dendrobium officinale* leaves by different drying methods.
