# Supplementary material for: Comparative Evaluation of the Physiochemical Properties, and Antioxidant and Hypoglycemic Activities of Dendrobium officinale Leaves Processed Using Different Drying Techniques
Source: Antioxidants (Basel). 2023 Oct 26;12(11):1911. doi: 10.3390/antiox12111911 (PMC10669270; doi:10.3390/antiox12111911)
Supplement: Supplementary file 1 [file antioxidants-12-01911-s001.zip › antioxidants-2633843-supplementary.pdf]

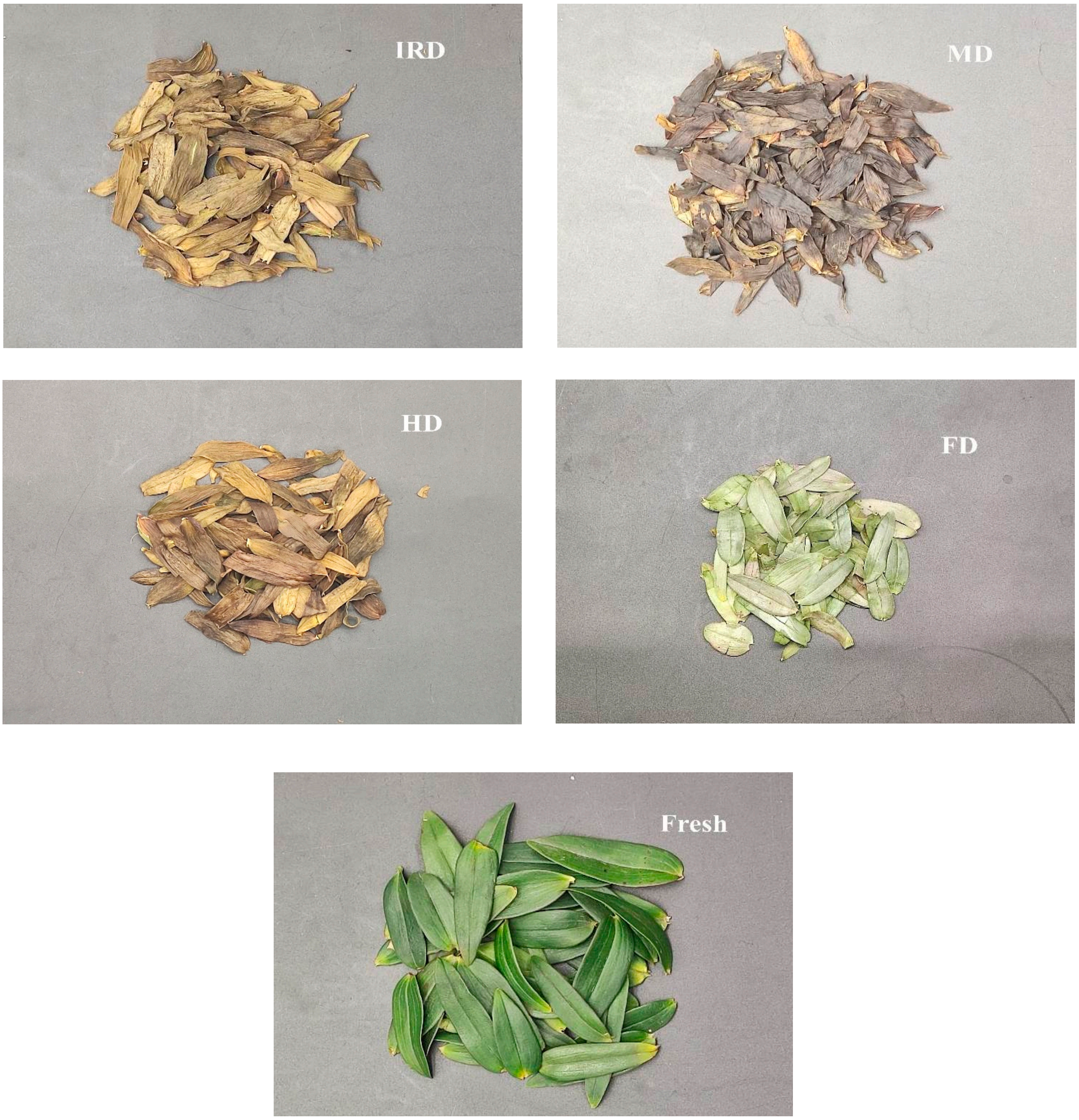

Figure S1. Photos of the fresh (F) and dried *Dendrobium officinale* leaves by different drying methods. FD, freeze drying; HD, hot air drying; SD, sun drying; MD, microwave drying.
